# Associated changes in stiffness of collagen scaffolds during osteoblast mineralisation and bone formation

**DOI:** 10.1186/s13104-022-06203-z

**Published:** 2022-09-24

**Authors:** Deniz Bakkalci, Auxtine Micalet, Rawiya Al Hosni, Emad Moeendarbary, Umber Cheema

**Affiliations:** 1 Division of Surgery and Interventional Science, Centre for 3D Models of Health and Disease, University College London (UCL), Charles Bell House, 43-45 Foley Street, W1W 7TS London, UK; 2 Department of Mechanical Engineering, University College London (UCL), Torrington Place, WC1E 6BT London, UK

**Keywords:** AFM, 3D models, Bone, Tissue engineering, Stiffness, ECM

## Abstract

**Objective:**

Engineering bone in 3D is important for both regenerative medicine purposes and for the development of accurate in vitro models of bone tissue. The changing material stiffness of bone tissue had not yet been monitored throughout the process of mineralisation and bone nodule formation by osteoblasts either during in vitro engineering or in development perspective.

**Results:**

Within this short research note, stiffness changes (Young’s modulus) during in vitro bone formation by primary osteoblasts in dense collagen scaffolds were monitored using atomic force microscopy. Data analysis revealed significant stiffening of 3D bone cultures at day 5 and 8 that was correlated with the onset of mineral deposition (p < 0.00005).

## Introduction

Bone tissue forms when differentiated bone cells, osteoblasts, deposit the appropriate matrix proteins, composed of an inorganic and organic matrix component. Through development or maturation of bone, the matrix component changes in structure and composition, thus altering the material properties of bone [1]. Osteoblasts synthesise bone matrix in two steps: (1) secreting collagen proteins (collagen type I) and non-collagen proteins including osteocalcin, osteonectin and bone sialoprotein II; (2) mineralisation and formation of the hydroxyapatite crystals. Hydroxyapatite crystals are formed through the deposition of mature apatite minerals. Furthermore collagen I is remodelled through increases in fibril diameter, orientation and cross-linking [2, 3].

Biophysical parameters, including stiffness, are essential to define the mechanical integrity of bone. The mechanical properties of fully formed bone are well defined [4, 5]. Even so, the changing material properties of developing bone are not well understood and have not been extensively studied. There is a significant relationship between tissue stiffness and mineralisation but no significant difference in tensile strength between foetal bone and bone from new-born animals [6].

The process of mineralisation and bone nodule formation by osteoblasts is of particular interest. Monitoring the resultant changes in stiffness of a collagen scaffold caused by the cellular processes of mineralisation through to bone formation is challenging, but of immense interest as we decipher the changing material properties of developing bone. Bone forming-cells, osteoblasts have been widely used for in vitro bone formation studies. It is possible to culture neonatal rodent calvarial osteoblasts which form mature bone nodule formation in vitro in 2D [7, 8]. This process is enhanced when culturing primary osteoblasts in dense collagen (10%) scaffolds [9]. Bakkalci et al. have shown that by culturing primary osteoblast cells in 3D dense collagen, there is a significant increase in mineralisation and formation of large bone nodules compared to 2D [9]. This short note focuses on mineralisation and bone nodule formation and the effect these processes have on the changing stiffness of a 3D collagen scaffold.

Stiffness measures the deformation of a material under certain applied force [10, 11]. Its usual measure is the elastic modulus, also called Young’s Modulus (*E*). The stiffness unit is determined from stress over strain, Pa or N/m^2^ [11]. In tissues, the stiffness is based on the composition of the extracellular matrix. The tissue stiffness of bone is significantly higher (at 15 MPa) compared to other tissues such as brain (1 kPa) and articular cartilage (6 kPa) [12]. Techniques to complete ex vivo measurements for mechanical characterisation include atomic force microscopy (AFM), microindentation, and shear rheometry [13–16]. AFM can be used for both single cell level and bulk tissue level mechanical measurements.

Mineralisation and maturation of the inorganic component of bone determines the high strength and stiffness of bone tissue, with collagen playing a minor component [17]. Herein, we utilised AFM to measure changes in the stiffness of an osteoblast seeded dense collagen scaffold during the processes of mineralisation through to bone nodule formation. We correlated the stage-specific genes associated with bone formation, to changes in stiffness measurements.

## Main text

### Materials and methods

#### Isolation of primary rat osteoblasts

The primary calvarial rat osteoblasts were isolated, passaged and characterised as described in [9, 18, 19]. The cells were cultured for 3 days until they have reached confluency prior to 3D set-up at 37 ^o^C, 5% CO_2_ in α-MEM (Gibco through Thermo Fisher Scientific, Loughborough, UK), supplemented with 10% Foetal Bovine Serum, 2 mM L-glutamine (Life Technologies). Different techniques were used to characterise osteoblasts and osteoblast-driven bone formation and these include analysis of osteoblast gene markers and Alizarin red staining as described in [9].

#### Fabrication of 3D bone model

The osteoblasts were seeded in 2D (control) or 3D soft collagen scaffolds (0.2% collagen), or dense collagen scaffolds (10% collagen) in 24-well plates [20]. 3D soft and dense collagen scaffolds were prepared as described in [9] (pages 3–10). A mix of 10X Minimal Essential Medium (MEM) (Sigma-Aldrich, Dorset, UK), monomeric type I collagen, (First Link, Birmingham, UK) and the neutralising agent composed of 17% of 10 Molar (M) NaOH (Sigma-Aldrich, Dorset, UK) and 83% 10 M HEPES buffer (Gibco through Thermo Fisher Scientific, Loughborough) was prepared. Finally, a cell suspension of 7 × 10^4^ cells/well was added to this mix. 1.3 ml of cell/mix with 7 × 10^4^ cells per well were polymerised for 15 min at 37 ^o^C. For dense collagens plastic compressed using 24-well RAFT absorbers (Lonza, Slough, UK), where for soft collagens the gels were left uncompressed.

#### Bone formation with bone morphogenic agents

Culture media was replaced with α-MEM supplemented with the bone morphogenic agents (BMA). BMA was composed of 2 mM β-glycerophosphate, 10 nM dexamethasone, and 50 µg/ml ascorbate (Sigma-Aldrich, Dorset, UK).

#### Characterisation of bone nodules

Brightfield images were taken using the Zeiss AxioObserver with Apotome0.2 instrument and software (Zeiss, Oberkochen, Germany). Observation of the black dots represented mineralisation based on the classification of Orriss et al. [7]. The nodules with sharp, defined margins were identified as bone nodules.

#### Alizarin Red Staining

Calcium deposition was detected using Alizarin red staining (Sigma-Aldrich, Dorset, UK) at days 5, 8 and 14. The samples were formalin fixed (Genta Medical, York, UK), then washed with ddH_2_O and incubated with Alizarin red stain for 30 min followed by washing with ddH_2_O.

#### Haematoxylin and eosin staining

Formalin-fixed 3D samples were processed and wax embedded (Leica Biosystems, Germany). Samples were sectioned (5 μm) using a microtome (Leica, Milton Keynes, UK) and oven baked at 64 ^o^C. The haematoxylin and eosin (H&E) staining was completed as described in [9].

#### RNA extraction, cDNA synthesis, qPCR

RNA extraction, cDNA synthesis and qPCR were completed as described in [9]. For each condition, a minimum of 3 replicates were made. The RNA was extracted using TRI Reagent and was subjected to the chloroform induced phase separation method [21], with quality and quantity determined using a Nano-Drop (Thermo Fisher Scientific, Loughborough, UK). The RNA was transcribed into cDNA using High-Capacity cDNA Reverse Transcription Kit (Applied Biosystems through Fisher Scientific, Loughborough, UK). The primer design for the mineralisation gene alkaline phosphatase *ALPL* and the osteocyte gene *E11* designed by Bakkalci et al., [9] were used. The target genes were amplified using iTaq Universal SYBR Green Supermix. For each 10 µl qPCR reaction, 20 ng sample and 0.2 µM primer concentration were used. The CFX96 Touch System (Bio-Rad, Watford, UK) was used to run 40 cycles for the reaction. The ∆CT and 2^−∆∆CT^ methods [22] were followed to assess the relative gene expression normalised to the reference gene *Glyceraldehyde 3-phosphate dehydrogenase (GAPDH)* [23].

#### Atomic Force Microscopy (AFM) for stiffness measurements

The 3D samples were tested with an AFM (CellHesion® 200, JPK, Germany) on day 1, 5, and 8 following previously published methodology [24]. Briefly, for each time point, three 3D bone models and three 3D acellular controls were measured. Measurements were performed at room temperature, in Leibovitz’s L-15 Medium, no phenol red (Gibco™ through Fisher Scientific, Loughborough, UK). A stiff cantilever of spring constant of approximately 2 N/m (RFESP-75, Bruker) with a glued glass bead of 50 μm in diameter (Cospheric, California, USA) was used to probe the samples. Each sample was probed along a 4 × 4 map of 1500 × 1500 μm leading to a total of 16 measurements per sample. The set force was 700 nN to ensure an indentation of 10 μm minimum (< 10% of total height of the model). The Hertz model was fitted to the collected force curves to determine the Young’s Modulus E, assuming a Poisson ratio of 0.5 [25]. Data for each time point was normalised to its corresponding 3D acellular control of either soft or dense collagen scaffolds, as to obtain percentage change (% change = (E-E_control_)/E_control_).

#### Height measurement

Day 21 bone nodules formed in 3D dense collagen were air-dried in a Petri dish for 24 h. Air-dried collagen layer was defined as the baseline during height measurement and the height relative to the collagen layer was taken measured by Keyence VHX-7000 Digital Microscope (Keyence, Osaka, Japan).

#### Statistical analysis

All statistical analyses were conducted by using GraphPad Prism 9 Software Inc., La Jolla, CA, US for each condition with minimum of 3 experimental repeats. Shapiro-Wilk test (n$\ge$3) or D’Agostino test (n$\ge$8) was used as normality test for all data sets. The appropriate statistical significance tests were selected depending on the result of the normality test. The data in the graphs were provided as mean $\pm$ standard error mean (SEM), and in the text as mean $\pm$ standard deviation (SD). P-value < 0.05 was taken as statistical significance.

## Results

### Bone nodules do not form in soft collagen scaffolds

**Fig. 1:**
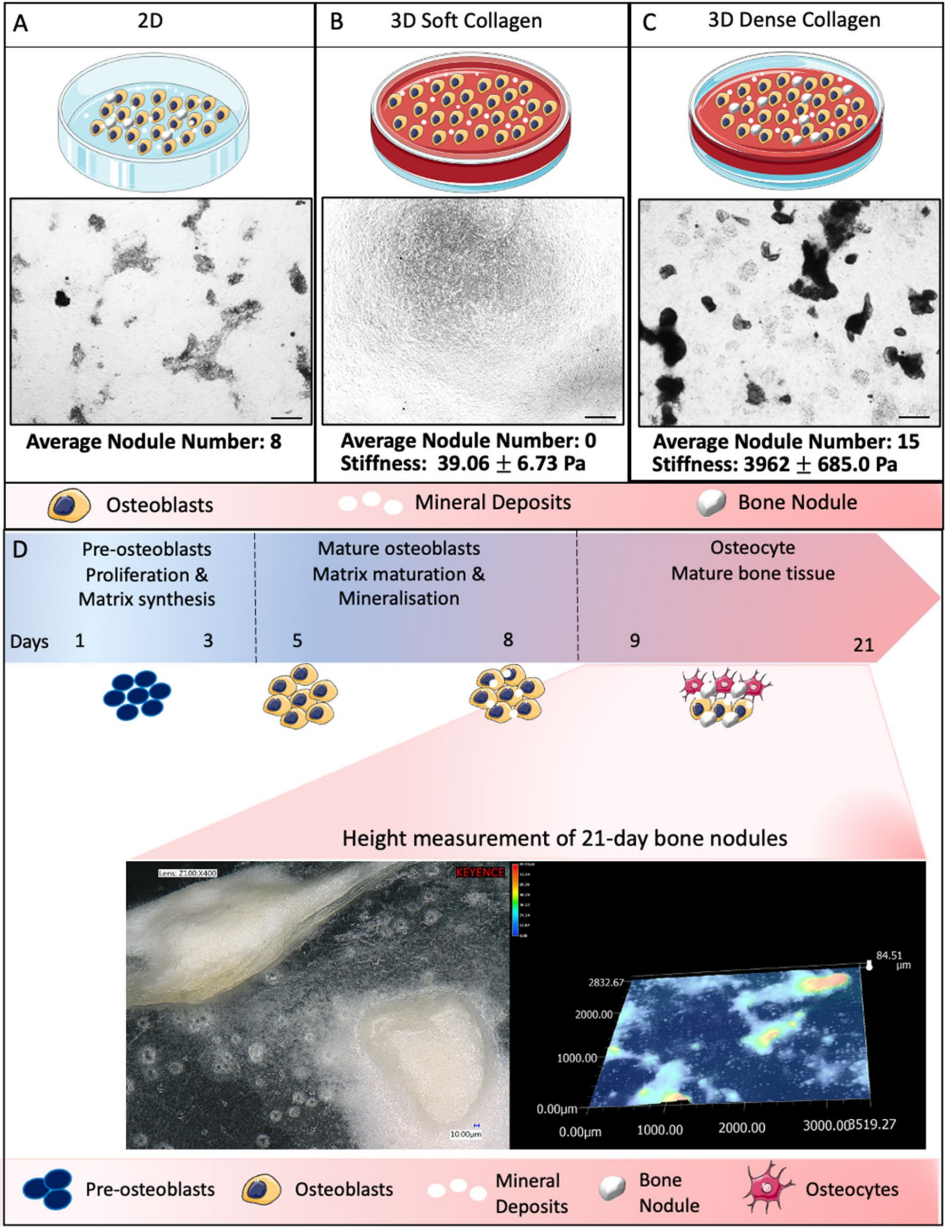
In vitro bone nodule formation. Bone formation in **a** 2D, **b** 3D soft collagen scaffold, and **c** 3D dense collagen scaffold at 14 of BMA application. 2.5× Magnification, Scale bar = 500 μm. **d** Bone nodule formation timeline in 3D dense collagen. Height measurement of 21-day old bone nodules by Keyence VHX-7000 Digital Microscope (Keyence, Osaka, Japan). Scale bar (left image) = 10 μm. The schematic has been created using SmartServier Medical Art

To support our previous finding on bone nodule formation in 3D dense collagen scaffolds, we seeded primary calvarial rat osteoblasts in 3D soft collagen scaffolds and in 3D dense collagen scaffolds for 21 days and bone nodule formation was compared with 2D control cultures (Fig. 1A). Osteoblasts did not deposit bone nodules in the soft collagen scaffolds, where the average stiffness was 39.06 ± 6.73 Pa (Fig. 1B). Mineralisation and hydroxyapatite crystals were significantly reduced in these soft collagen scaffolds compared to 2D and 3D dense collagen scaffolds (Fig. 1A and C). The average number of bone nodules in 3D dense collagen scaffolds was higher compared to 2D culture (Fig. 1A and C). The timeline of the bone nodule formation in 3D dense collagen scaffolds is described in Fig. 1D. The matrix maturation and mineralisation were visible by day 5, where the nodule formation began by day 8. Height measurement images show defined edges of the bone nodules and the nodule protrusion from the 3D dense collagen (Fig. 1D).

### Mineralisation increases the overall matrix stiffness of engineered bone in 3D dense collagen scaffolds

**Fig. 2:**
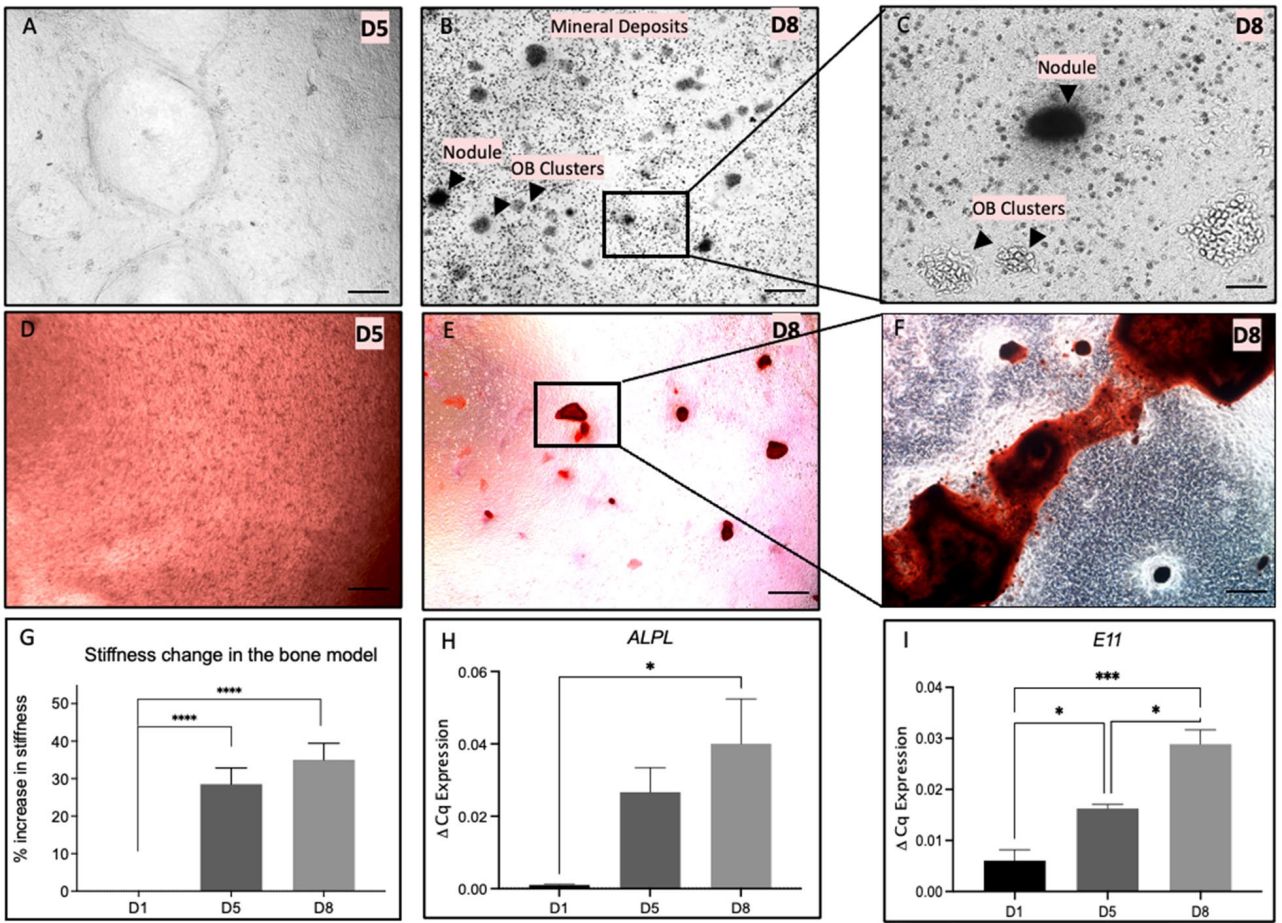
In vitro bone nodule formation in 3D dense collagen scaffolds.** a** Mineral deposition at day 5, Scale bar = 500 μm. **b** Osteoblast clusters and mineral and bone nodule deposition at day 8, Scale bar = 500 μm. **c** Bone nodule formation at day 8, Scale bar = 100 μm. Alizarin red stained 3D dense collagen scaffolds at **d** day 5 and **e** day 8. **f** Alizarin red stained 3D dense collagen scaffolds at day 8, Scale bar = 25 μm. **g** Percentage change in stiffness (%) at day 1, 5, and 8. Expression of **h** ALPL, and **i** E11 at days 1, 5, and 8. One-Way ANOVA, Dunnet’s Post Hoc; p-values 0.05< *, 0.005<**, 0.005<*** and 0.00005<****

Previous work has demonstrated that culturing primary osteoblasts in 3D dense collagen results in earlier and more extensive mineralisation and bone nodule formation [9]. The impact of mineralisation and bone nodule formation on the increasing stiffness of collagen scaffolds was measured using AFM. Mineralisation starts on day 5 in dense 3D scaffolds (Fig. 2A), confirmed by using alizarin red staining (Fig. 2D). By day 8, it was possible to visualise dense mineral deposits, osteoblast clusters and small bone nodules (Fig. 2B and C). The cuboidal mononuclear appearance of osteoblasts and mineral deposits was detected by day 8 (Fig. 2C). The sharp edges of the bone nodules were indicative of bone nodule formation and were verified by alizarin red staining (Fig. 2E). The alizarin red staining confirmed the formation of large bone nodules (Fig. 2F).

The average stiffness of an acellular 3D dense collagen scaffold was 3962 ± 685.0 Pa. With the onset of mineral deposition, the stiffness increased by 30% on day 5 (p < 0.00005) and by 35% on day 8 (p < 0.00005) compared to day 1 (Fig. 2G). The average stiffness of the 3D bone model at day 5 was 5473 ± 2725 Pa and at day 8 was 5700 ± 1087 Pa taken as an average over a defined 1500 × 1500 μm area.

The osteoblast and mineralisation marker *ALPL* [26] was upregulated 39-fold from day 1 to day 8 (p < 0.05) (Fig. 2H). The osteocyte marker E11 [27] increased 2.7-fold at day 5 (p < 0.05) and 4.8-fold at day 8 (p < 0.05) (Fig. 2I).

## Discussion

The culture of primary osteoblasts in 3D dense collagen scaffolds results in significant mineralisation and bone nodule formation compared to cultures in 2D and soft collagen scaffolds. It is not surprising that the more biomimetic the collagen type I environment (dense) enhances the osteoblast-driven bone formation considering that collagen I is the main organic ECM protein found in bone [28, 29].

Herein are three novel findings on understanding how stiffness changes during bone formation. The first is that stiffness of the dense collagen scaffold itself is not impacted by the addition of bone morphogenic reagents. The stiffness of the dense collagen was ~ 4 kPa on day 1, compared to soft collagen scaffolds of ~ 40 Pa, indicating that the higher range of stiffness required to initiate bone matrix deposition. The stiffness changes as the tissue develops and therefore the active process of mineralisation on day 5 concords with a significantly increased stiffness observed on the same day (30% increase). The bone nodules were formed on day 8, when stiffness increased by 5% compared to day 5, suggesting a stiffness threshold that should be achieved in vitro to initiate mineralisation and bone formation. After reaching the stiffness threshold, the increase in stiffness is more limited. The small increase in stiffness percentage change and large standard deviation can be explained by the averaging method used. AFM measures were taken every 375 μm (1500 × 1500 μm area), and then averaged, thus minimising biased results, and avoiding solely focusing on mineral points and bone nodules. Instead, both acellular and mineralised areas of a sample are measured at random, leading to the average stiffness increasing with the frequency of minerals and nodules per sample.

This work is the first to quantify in vitro bone nodule formation and ossification by osteoblasts with increasing tissue stiffness. We have optimised an active bone forming model in 3D dense collagen and characterised how stiffness changes prior to and during the formation of bone nodules. This model closely represents the native starting reference for bone formation, and it allows a platform to study pathological alterations related to bone diseases.

## Limitations

- Bone nodules form randomly throughout the culture and due to consistent AFM measurements for every sample, it may be possible to miss the nodules and mineralisation.

## Data Availability

The authors confirm that data and material in this study are presented in main manuscript.

## Abbreviations

AFM: Atomic force microscopy
BMA: Bone morphogenic agents
H&E: Haematoxylin & Eosin
*GAPDH*: *Glyceraldehyde 3-phosphate dehydrogenase*
MEM: Minimal essential medium
qPCR: quantitative polymerase chain reaction
